# The Global Status of Research in Ankle Fracture: A Bibliometric and Visualized Study

**DOI:** 10.3389/fsurg.2022.853101

**Published:** 2022-03-14

**Authors:** Jianshuang Zeng, Cheng Xu, Gaoxiang Xu, Daofeng Wang, Wupeng Zhang, Hua Li, Xuewen Gan, Ying Xiong, Jiantao Li, Licheng Zhang, Peifu Tang

**Affiliations:** ^1^School of Medicine, Nankai University, Tianjin, China; ^2^Senior Department of Orthopedics, The Fourth Medical Center of Chinese PLA General Hospital, Beijing, China; ^3^National Clinical Research Center for Orthopedics, Sports Medicine and Rehabilitation, Beijing, China; ^4^Department of Orthopedics, Kunming Medical University, Yanan Hospital, Kunming, China

**Keywords:** ankle fracture, bibliometric, visualization studies, co-authorship analysis, co-citation analysis

## Abstract

**Background:**

Ankle fractures are common lower extremity fractures that pose a significant economic and social burden. This study analyzed the ankle fracture research literature between 2000 and 2021 to clarify the current status of ankle fracture research and predict future research trends.

**Methods:**

Publications related to ankle fractures published between 2000 and 2021 were retrieved from the Web of Science Core Collection. Then Bibliometric analysis and Visualized Study were performed by VOSviewer software.

**Results:**

A total of 2656 publications were retrieved. The number of publications related to ankle fractures is increasing every year. The top countries and journals in terms of the total number of publications, number of citations, and H-index ranking were USA and foot and ankle int. Lorich DG had the most publications in this field. University of Amsterdam's research group had the biggest number of publications in this field. Co-occurrence analysis clustered the keywords into seven clusters: survival analysis and prognosis study, internal fixation treatment study, treatment study of combined deltoid ligament rupture, treatment study of combined inferior tibiofibular ligament injury, treatment study of posterior ankle fracture, treatment study of postoperative traumatic arthritis of ankle fracture, and treatment study of ankle injury in children.

**Conclusions:**

The importance of ankle fractures is increasing year by year with the aging process, and the number of publications related to ankle fractures will not continue to increase in the future. Survival and prognosis studies, internal fixation studies, combined deltoid ligament rupture studies, and combined inferior tibiofibular ligament injury studies may become the future research hotspots in the field of ankle fractures.

## Introduction

Ankle fractures, accounting for 3.9–10.2% of adult fractures, are the most common type of fracture of the lower extremity (1). The incidence of ankle fractures is increasing with the socioeconomic and aging process of the population (2). The occurrence is often found in physical exercise, strenuous labor, and other activities. Due to the complexity of the ankle joint and the variety of factors that cause injuries, ankle fractures can take many different forms in clinical practice, and improper management can lead to poorly matched and unstable ankle joints, resulting in complications such as ankle arthritis, deformity healing, and dysfunction (3). In recent decades, many scholars have reported a large number of studies on the diagnosis and treatment of ankle fractures, which are important information for the progress of ankle fracture diagnosis and treatment.

Bibliometric, a subdiscipline of library and information science, enables qualitative and quantitative assessment of research trends based on the bibliometric characteristics of bibliographic databases and the relevant contributions of journals, institutions, and countries (4). To further clarify the current state of research and research trends in ankle fractures and to provide evidence for the development of more rational medical measures for the prevention and treatment of ankle fractures. This study used bibliometric methods to analyze the publications related to ankle fractures since 2000 and generated a visual plot to visualize the current status and hotspots of research in this field, to explore future trends, and to provide directional guidance and new ideas for future research on ankle fractures.

## Materials and Methods

### Data Sources and Search Strategy

The data were all publicly available and did not require the approval of the Institutional Review Board. Our research methodology is similar to previous bibliometric studies (5–8). Data were obtained from the Web of Science Core Collection, by searching for publications in English between 2000.01.01 and 2021.12.01 (9). The following search terms were used: ankle fracture^*^, ankle joint fracture^*^, fracture^*^ of the ankle, fracture^*^ to the ankle, fracture^*^ of the ankle joint, fracture^*^ to the ankle joint. The search terms for randomized controlled trials (RCTs) were: ankle joint fracture^*^, fracture^*^ of the ankle, fracture^*^ to the ankle, fracture^*^ of the ankle joint, fracture^*^ to the ankle joint, and randomized controlled trial NOT meta^*^.

### Data Screening and Collection

#### Inclusion Criteria

Articles, conference abstracts, reviews, correspondence, conference articles, editorial material, research notes, errata.

#### Exclusion Criteria

Literature not relevant to this study, unpublished literature.

#### Extracted Information

Publication year, country, number of publications, total citations, average citations per term, H-index, journal, author, institution, and research fund direction.

### Bibliometric Analysis

Microsoft Office Excel 2016 (Microsoft Corporation, Santa Rosa, CA, USA) was used to rearrange and combine the extracted literature in terms of publication year, country, number of publications, total citations, average citations per term, H-index, journal, author, institution, and research fund direction. The basic characteristics of the data were analyzed to generate statistical charts and perform quantitative and qualitative analysis on the statistical charts. The Hi-index, also known as the h-factor, is a new method of evaluating academic achievement and can be an important indicator of a researcher's research-level or measure the impact of a journal or institution (10, 11). The Trend Line function in Microsoft Office Excel 2016 was used to build the predicted publication growth model equation as well.

### Visualized Study

VOSviewer version 1.6.17 (Leiden University Center for Science and Technology Studies, Leiden, the Netherlands) was used to complete co-citation analysis, co-authorship analysis, co-occurrence analysis, and bibliographic coupling analysis and present the results in a graphical format. The associations between authors, institutions, and countries/regions were visualized using weighted total link strength (TLS) lines.

The TLS represents the degree of association between different items, and a larger value means a higher degree of association. In the visual graph, the thickness of the line is used to represent the size of the TLS value.

Co-citation analysis was proposed by Small (12), which takes the literature as the subject of analysis and uses multivariate statistical analysis methods such as cluster analysis to reduce the inter-citation relationships between the subjects of analysis to a relatively small number of categories and present them graphically as a process. This study uses VOSviewer software to calculate and graphically present the co-citation link strength between different items.

Co-occurrence analysis was first proposed by Callon M (13), by counting and hierarchically clustering the number of occurrences of different items in the same literature, revealing the degree of association between items, and thus clarifying the changes in research directions and hotspots represented by a different item. We use VOSviewer to quantify and graphically display the different classifications and determine future research trends based on this.

Bibliometric coupling firstly proposed by Kessler MM (14) is a more advanced method of literature coupling research. If two articles A and B cite the same literature, there is a coupling between them, indicating that the two articles have similar research content. We use VOSviewer to quantify and graphically display the coupling between the literature.

## Results

### Trends of Global Publication

#### Number and Trend of Publications

**Figure 1:**
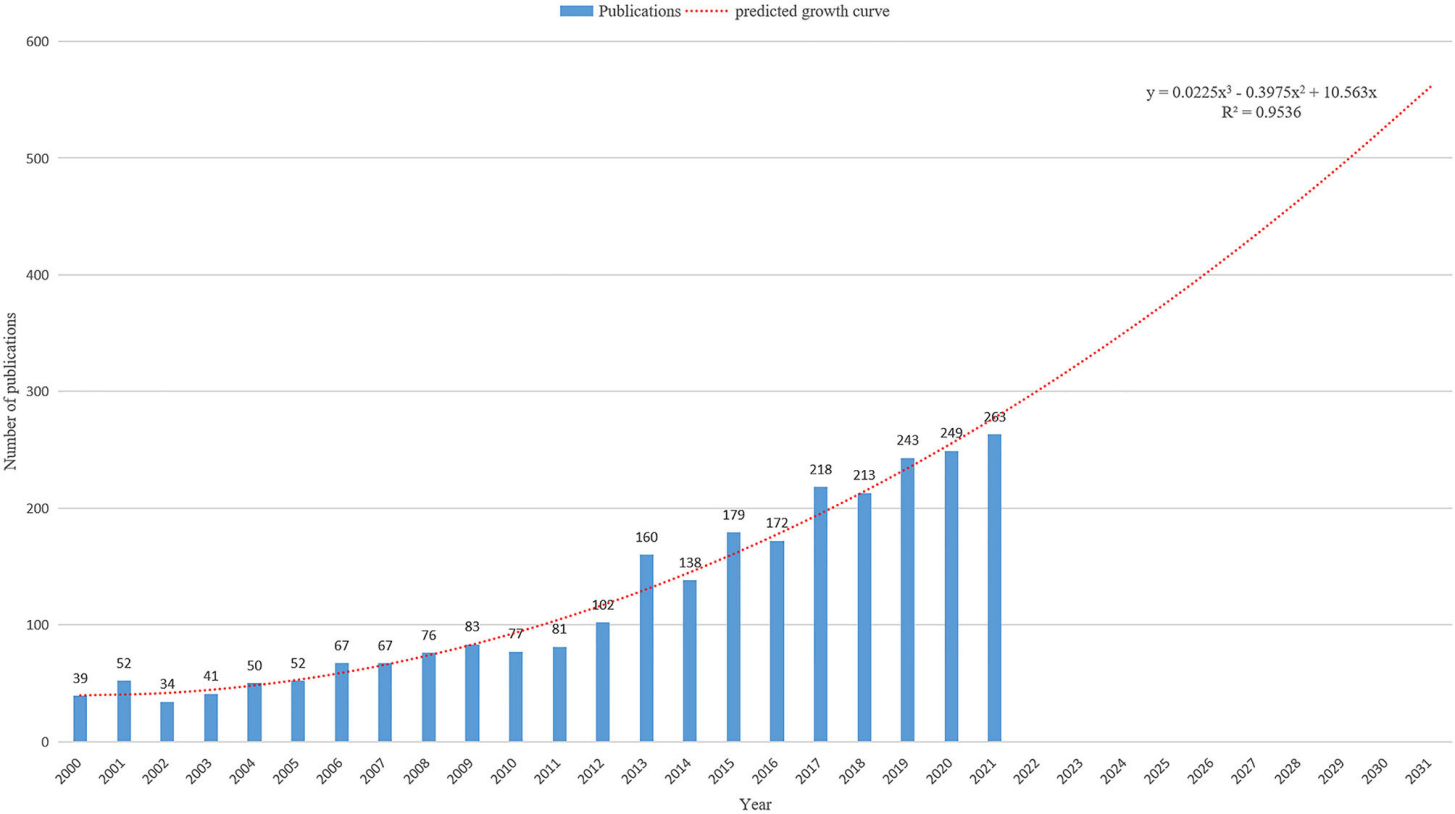
The number of publications by year and the predicted growth curve of ankle fractures.

**Figure 2:**
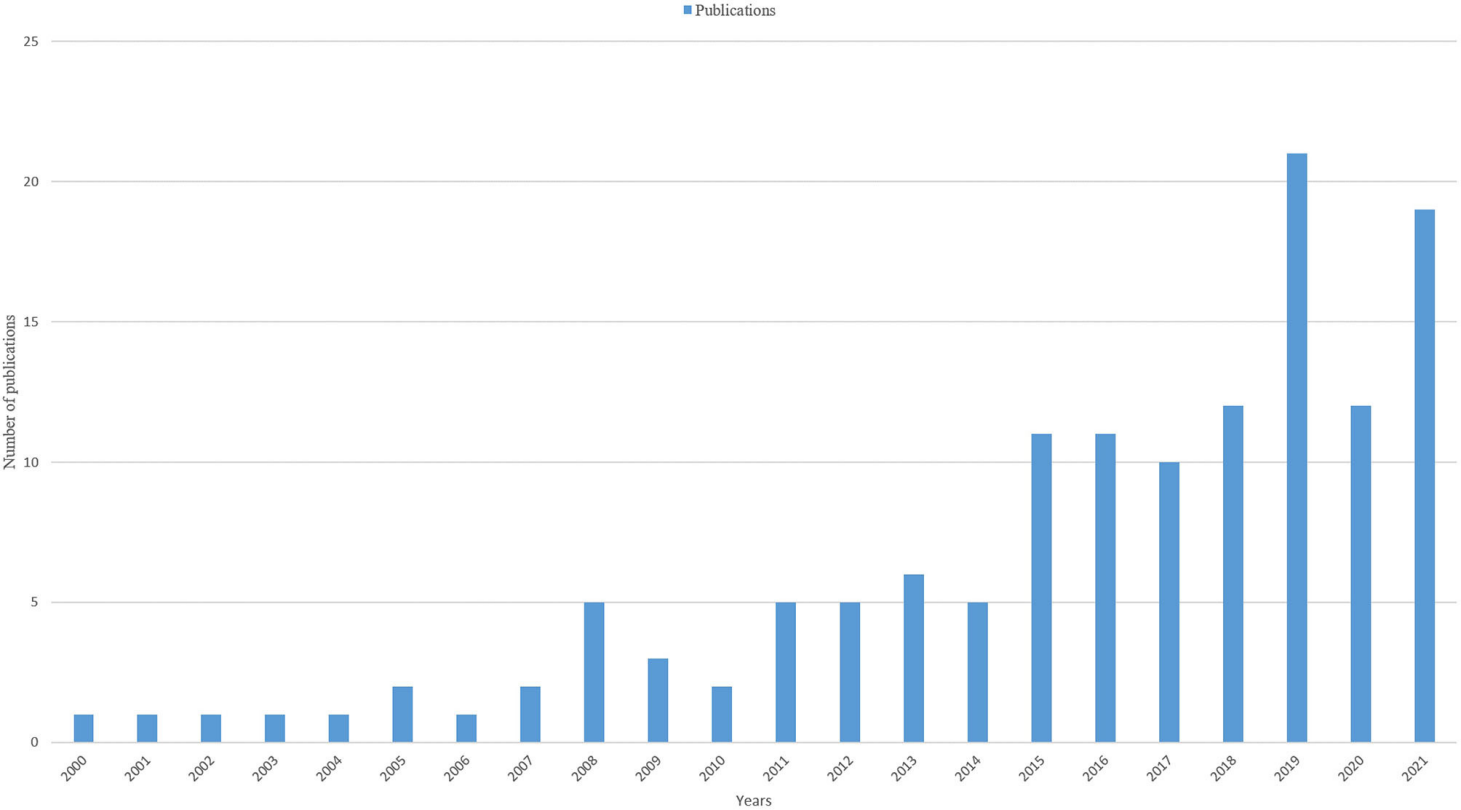
The number of publications of RCTs by year.

Since 2020-2021, there was an overall upward trend in the number of ankle fracture-related publications (Figure 1), with the highest number of publications published in 2021 (*n* = 263; 9.9%). The predicted publication growth model equation based on historical data was y = 0.0225x^3^-0.3975x^2^ + 10.563x, R^2^ = 0.9536, with x representing the year and y representing the predicted number of publications per year. According to this equation, the number of publications in this field is expected to exceed 600 in 10 years. The total number of articles published in RCTs from 2001 to 2021 is 137, with the most number of publications in 2019 (*n* = 21; 15.3%) (Figure 2).

### Contribution of Each Country/Region

**Figure 3:**
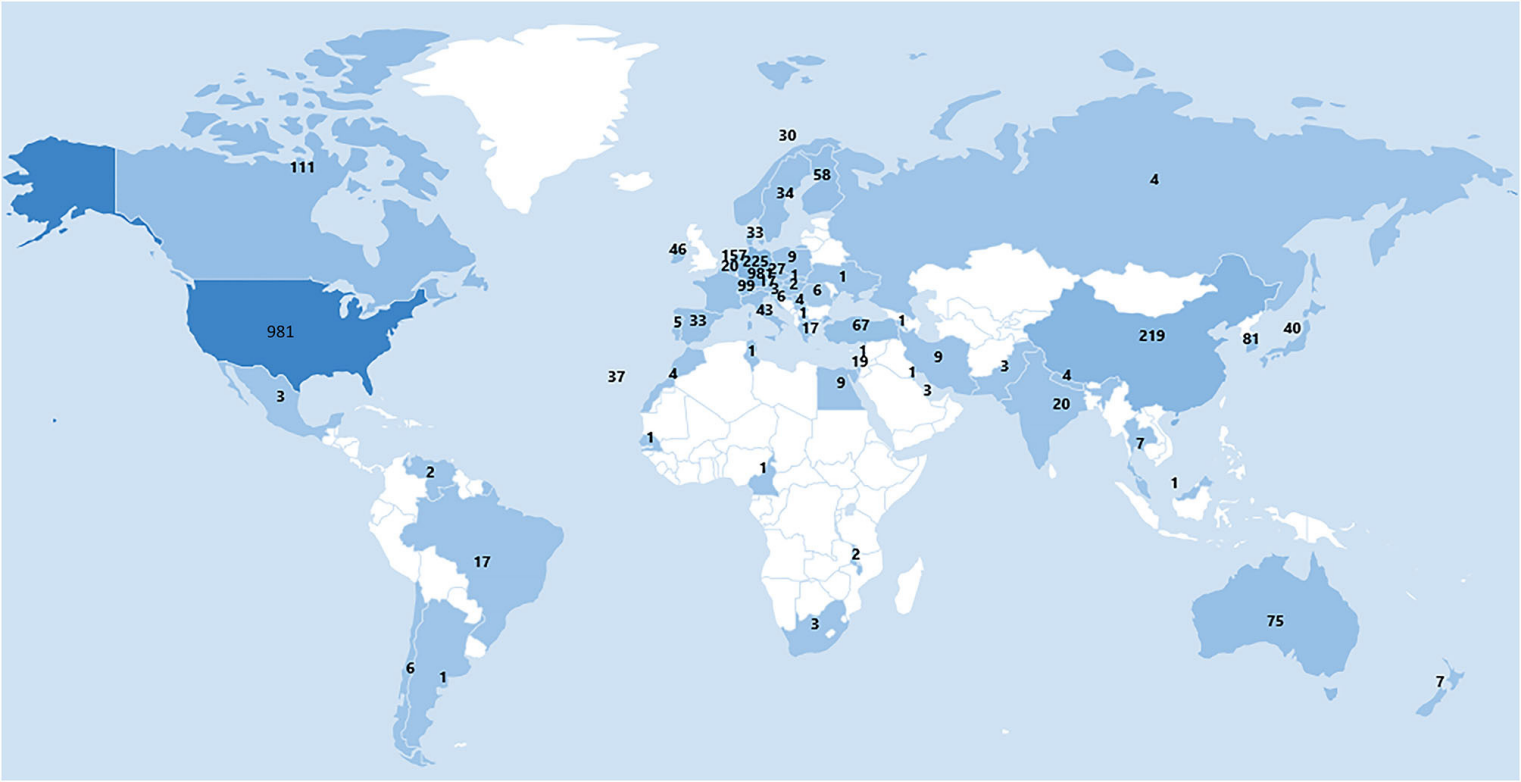
Distribution map of publications in different country/region.

A total of 66 Countries/Regions contributed published articles regarding ankle fractures (Figure 3). The United States had the highest number of publications in this area (*n* = 981; 36.9%), followed by England (*n* = 303; 11.4%), Germany (*n* = 225; 8.5%), China (*n* = 219; 8.25%) and the Netherlands (*n* = 157; 5.91%).

### Quality of the Publications of Each Country/Region

**Figure 4:**
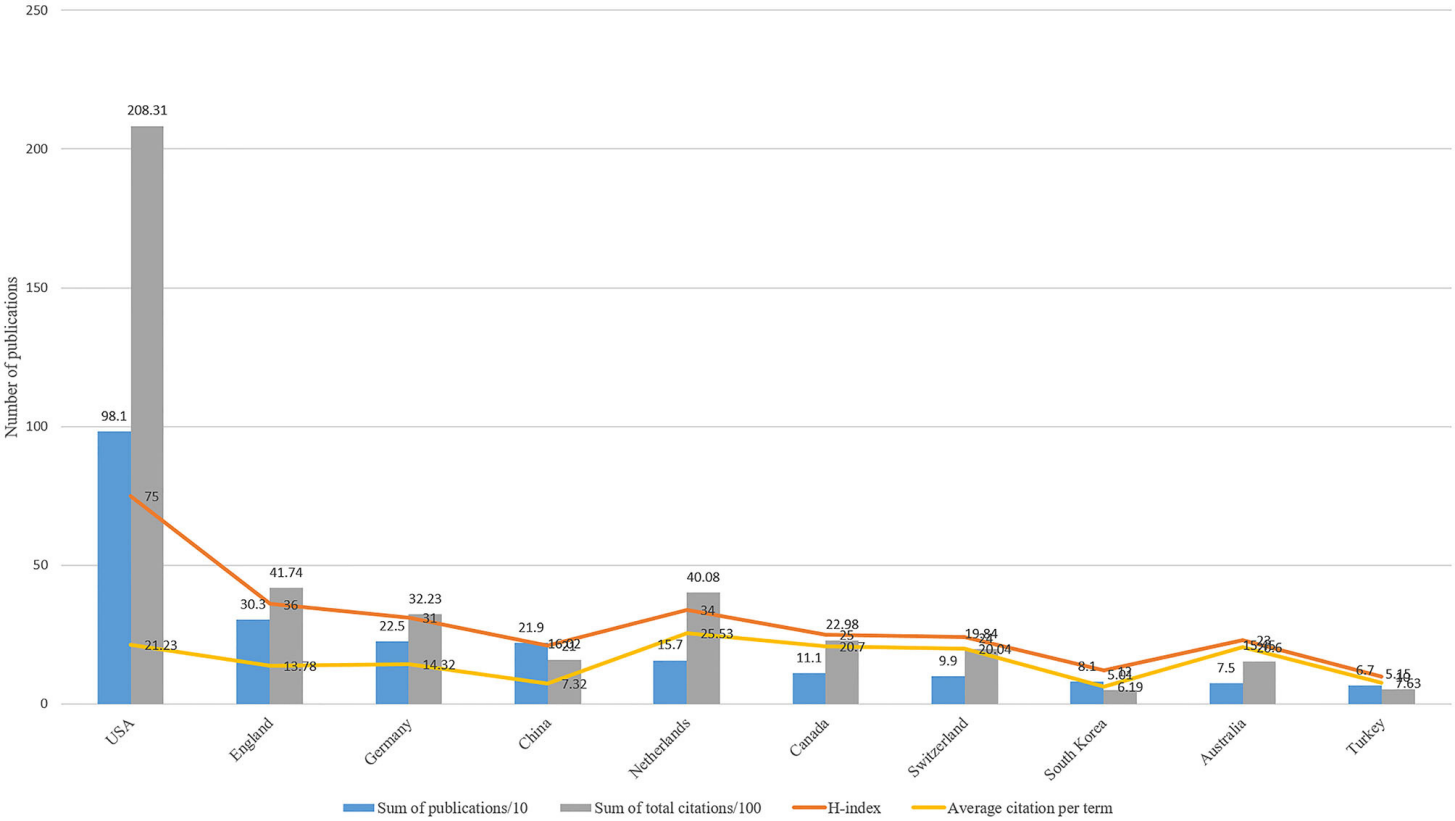
The top 10 productive and cited country/region in ankle fracture research.

The United States had the highest H-index (H-index = 75), followed by England (H-index = 36), Germany (H-index = 31), and China (H-index = 21). The United States also had the most total citations (*n* = 20,831), followed by the England (*n* = 4,174), the Netherlands (*n* = 4,008), Germany (*n* = 3,223), and Canada (*n* = 2,298). The publications from the Netherlands had the highest average citation per term (*n* = 25.53), followed by the USA (*n* = 21.23), Australia (*n* = 20.6), and Switzerland (*n* = 20.04) (Figure 4).

### Analysis of Global Publication Trends

#### Journals

**Figure 5:**
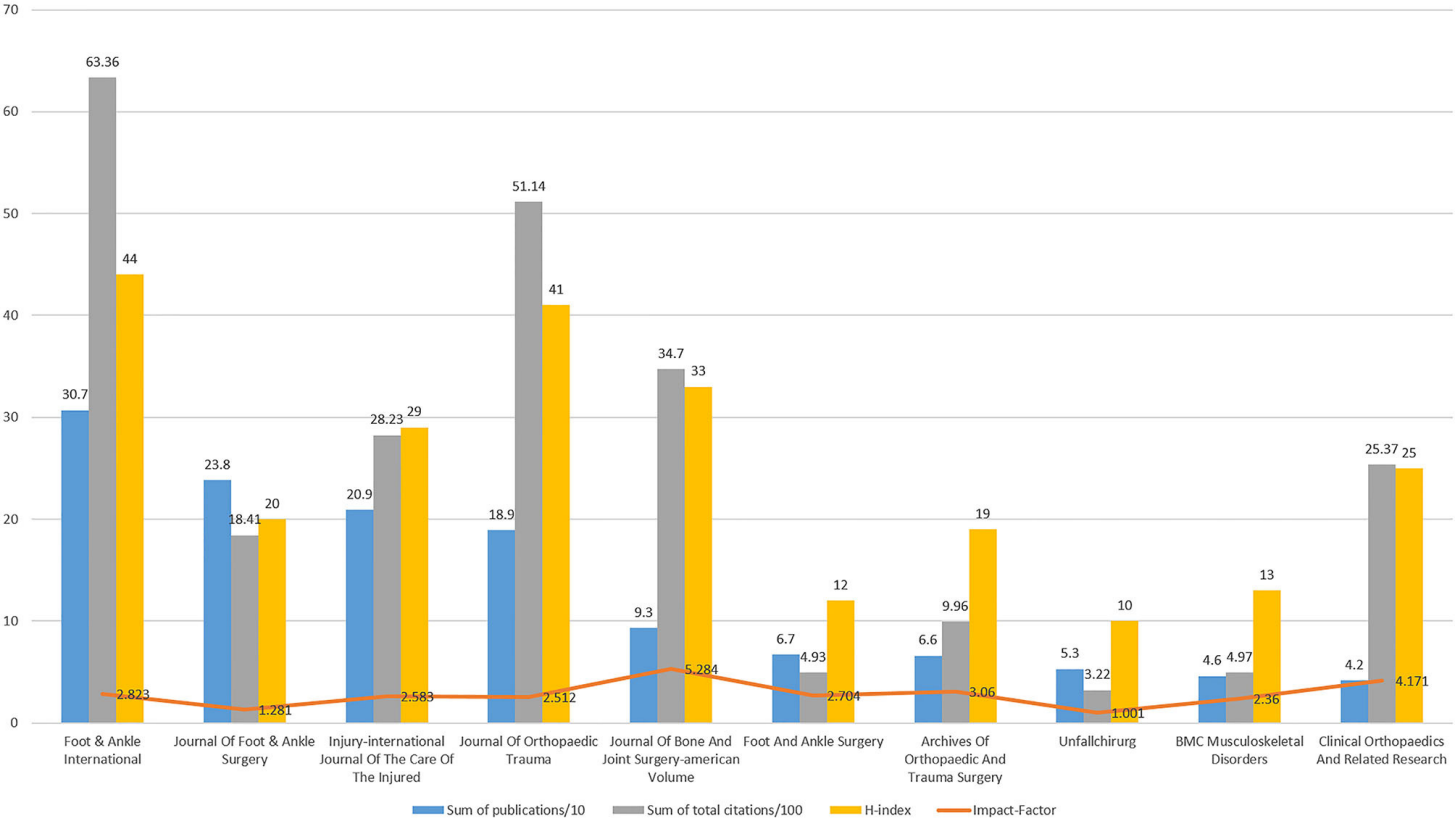
The top 10 productive and cited journals in ankle fracture research.

*Foot* & *Ankle International* had the most publications regarding ankle fractures (*n* = 307, 11.56%), followed by *Journal Of Foot* & *Ankle Surgery* (*n* = 238, 8.96%), *Injury-international Journal Of The Care Of The Injured* (*n* = 209, 7.87%), and *Journal Of Orthopedic Trauma* (*n* = 189, 7.11%), Journal Of Bone And Joint Surgery -American Volume (*n* = 93, 3.50%). *Foot* & *Ankle International* also had the highest number of citations (*n* = 6,336), followed by the *Journal Of Orthopedic Trauma* (*n* = 5,114), *Journal of Bone and Joint Surgery-American Volume* (*n* = 3,470), and *Injury-international Journal Of The Care Of The Injured* (*n* = 2,823). *Clinical Orthopedics and Related Research* were *n* = 2,537. *Journal Of Bone And Joint Surgery-American Volume* had the highest impact factor (IF = 5.284), followed by *Clinical Orthopedics and Related Research* (IF = 4.171) (Figure 5).

#### Research Areas

**Figure 6:**
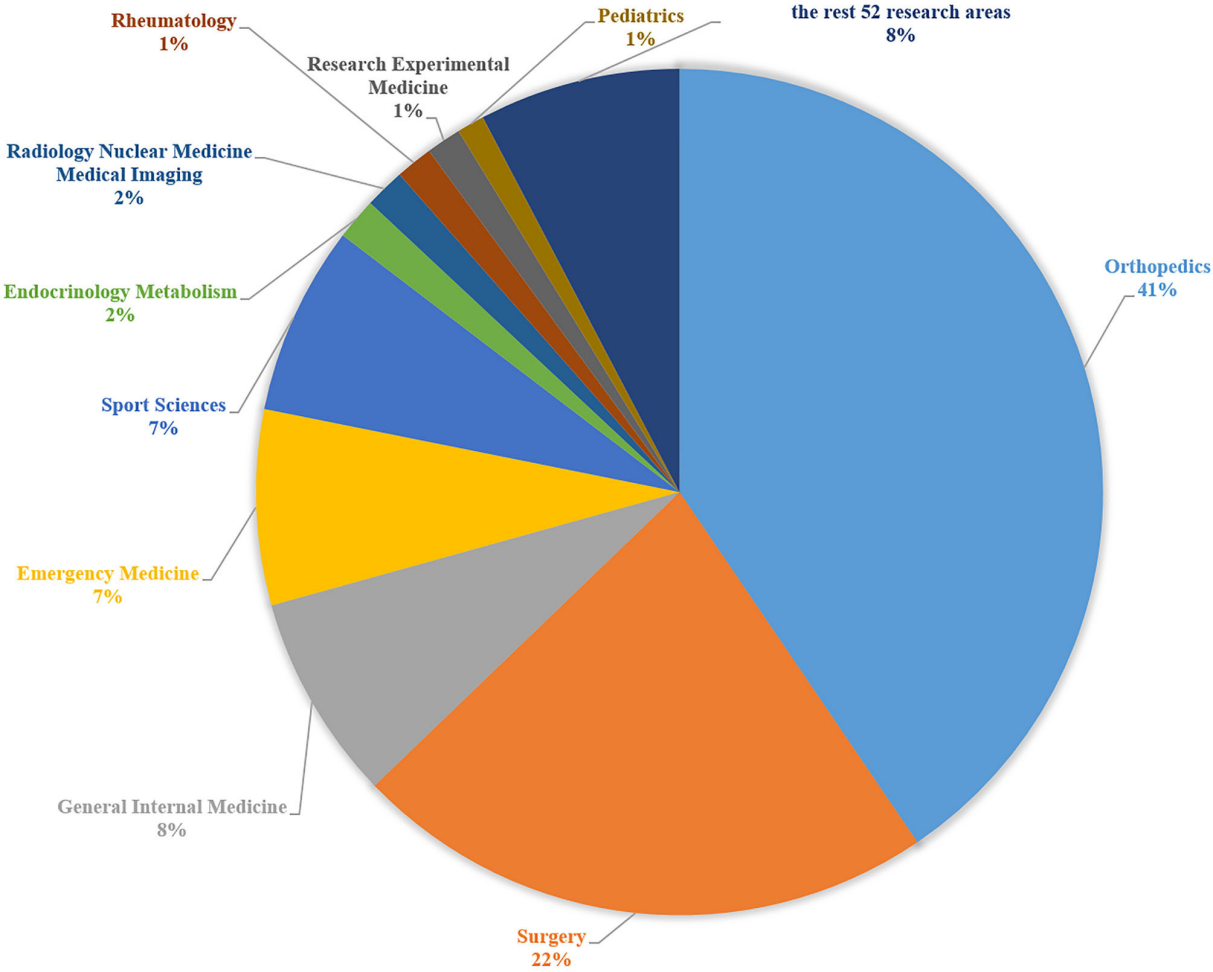
The top 10 research directions of ankle fractures.

Of the 62 research areas, the top 10 are Orthopedics, Surgery, General Internal Medicine, Emergency Medicine, Sport Sciences, Endocrinology Metabolism, Radiology Nuclear Medicine Medical Imaging, Rheumatology, Research Experimental Medicine, Pediatrics (Figure 6).

#### Authors

The ten authors with the biggest number of publications are Lorich DG (*n* = 38), Rammelt S (*n* = 36), Egol KA (*n* = 32), Schepers T (*n* = 32), Kwon JY (*n* = 28), Helfet DL *n* = 24), Ornetta P (*n* = 22), Hoogendoorn JM (*n* = 19), Van Den Bekerom MPJ (*n* = 19), Van Dijk CN (*n* = 19) (Table 1).

**Table 1:** The top 10 productive and cited authors in ankle fracture research.

**Authors**	**Publications**	**Citations**	**Average per item**	**H-index**	**Country/Region**	**Institution**
Lorich DG	38	1,526	40.16	17	USA	Hospital for Special Surgery
Rammelt S	36	553	15.36	13	Germany	University Hospital Carl Gustav Carus
Egol KA	32	1,060	33.13	18	USA	NYU-Hospital for Joint Diseases
Schepers T	32	735	22.97	14	Netherlands	University of Amsterdam
Kwon JY	28	180	6.43	7	USA	Massachusetts General Hospital
Helfet DL	24	1,259	52.46	16	USA	Hospital for Special Surgery
Tornetta P	22	609	27.68	15	USA	Boston University Medical Center
Hoogendoorn JM	19	287	15.11	10	Netherlands	Haaglanden Medical Center
Van Den Bekerom MPJ	19	610	32.11	14	Netherlands	Onze Lieve Vrouwe Gasthuis Hospital
Van Dijk CN	19	791	41.63	13	Netherlands	University of Amsterdam

#### Institutions

The top ten institutions with the biggest number of publications are University of Amsterdam, Harvard University, Hospital for Special Surgery, Academic Medical Center Amsterdam, University of Texas System, University of California System, Pennsylvania commonwealth system of higher education, New York University, Technische Universitat Dresden, Massachusetts General Hospital, and the above 10 institutions located in Western countries (7 in the USA, 2 in the Netherlands and 1 in Germany) (Table 2).

**Table 2:** The top 10 productive and cited institutions in ankle fracture research.

**Institutions**	**Publications**	**% of total publications**	**Country/ Region**	**Citations**	**Average per item**	**H-index**
University of Amsterdam	67	2.523	Netherlands	1,772	26.45	22
Harvard University	64	2.41	USA	1,011	15.8	17
Hospital for Special Surgery	56	2.108	USA	1810	32.32	20
Academic Medical Center Amsterdam	54	2.033	Netherlands	1,690	31.3	22
University of Texas System	53	1.995	USA	884	16.68	14
University of California System	46	1.732	USA	924	20.09	15
Pennsylvania commonwealth system of higher education	45	1.694	USA	1,142	25.38	15
New York University	42	1.581	USA	1,310	31.19	20
Technische Universitat Dresden	40	1.506	Germany	628	15.7	13
Massachusetts General Hospital	39	1.468	USA	655	16.79	14

#### Funding Organizations

United States Department of Health Human Services funded the most publications (*n* = 54; 2.0%), followed by National Institutes of Health (*n* = 51; 1.92%) and National Natural Science Foundation of China (*n* = 31; 1.167%). Six of the top ten funding agencies are located in the United States (Table 3).

**Table 3:** The top 10 productive and cited funding agencies in ankle fracture research.

**Funding agencies**	**Publications**	**% of total publications**	**Country/** **Region**
United States Department of Health Human Services	54	2.033	USA
National Institutes of Health	51	1.92	USA
National Natural Science Foundation of China	31	1.167	China
National Institute For Health Research	22	0.828	UK
National Institute of Arthritis Musculoskeletal Skin Diseases	18	0.678	USA
Stryker	18	0.678	USA
European Commission	15	0.565	Europe
Amgen	12	0.452	USA
National Health And Medical Research Council of Australia	12	0.452	Australia
Synthes	12	0.452	USA

### Co-authorship Analysis

#### Authors

A total of 177 authors had at least five publications. Lorich DG had the highest number of TLS (TLS = 101), followed by Helfet DL (TLS = 71), Garner MR (TLS = 55), Schottel PC (TLS = 52), and Pakarinen H (TLS = 51).

#### Institutions

A total of 207 institutions had at least five publications. The five institutions with the highest number of TLS were hospital special surgery (TLS = 56), University Toronto (TLS = 46), Massachusetts general hospital (TLS = 39), University Pittsburgh (TLS = 39), McMaster University (TLS = 33).

#### Countries/Regions

A total of 39 countries have at least five publications. The five countries with the highest number of TLS markings are the United States (TLS = 176), Germany (TLS = 101), England (TLS = 96), Canada (TLS = 83), and Netherlands (TLS = 69).

### Bibliometric Coupling Analysis

#### Authors

A total of 177 authors have at least five publications. The five authors with the highest number of TLS are Lorich DG (TLS = 43,945), Pakarinen H (TLS = 38,649), Flinkkila T (TLS = 30,109), Helfet DL (TLS = 29,442), Rammelt S (TLS = 27,264).

#### Journals

A total of 80 journals had at least five publications. The five journals with the highest number of TLS were Foot & Ankle International (TLS = 1,886,638), Journal of Foot & Ankle Surgery (TLS = 125,743), Journal of Orthopedic Trauma (TLS = 120, 213), Injury-international Journal of The Care Of The Injured (TLS = 118,406), Journal of Bone And Joint Surgery-American Volume (TLS = 54,472).

#### Institutions

A total of 207 institutions have at least five publications. The five institutions with the highest number of TLS are Hospital for Special Surgery (TLS = 52,803), University of OULU (TLS = 38,450), Academic Medical Center Amsterdam (TLS = 27,491), University of Amsterdam (TLS = 26,765), New York Presbyterian Hospital (TLS = 23,127).

#### Countries/Regions

A total of 39 countries have at least five publications. The five countries with the highest number of TLS are the USA (TLS = 421,803), Germany (TLS = 160,733), Netherlands (TLS = 147,100), England (TLS = 143,517), and China (TLS = 119,532).

### Co-citation Analysis

#### Journals

**Figure 7:**
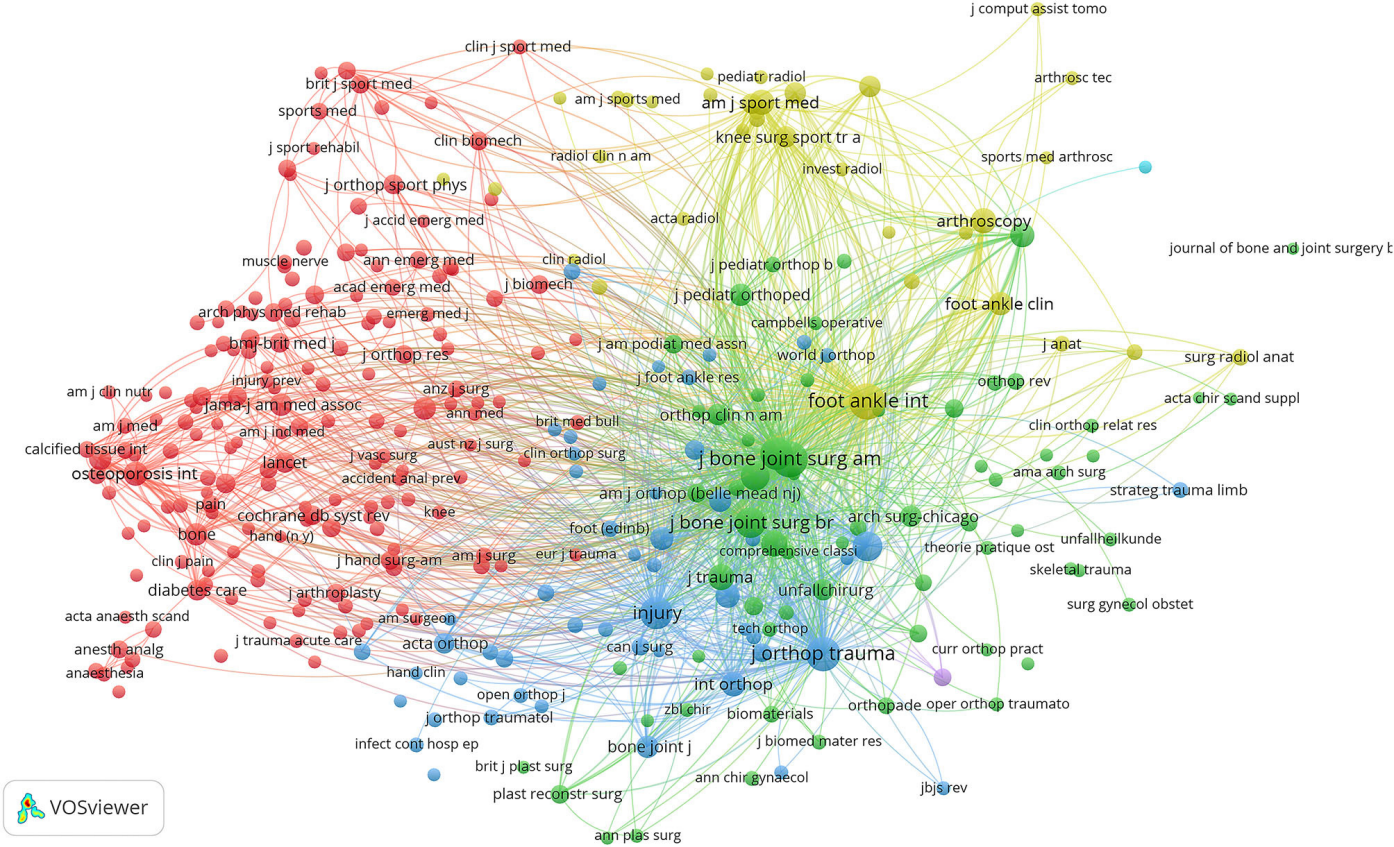
Co-citation analysis map of journals on ankle fracture.

There were 322 journals cited at least 20 times. The five journals with the highest number of TLS in order were Foot & Ankle International (TLS = 208,110), Journal of Bone And Joint Surgery-American Volume (TLS = 191,519), Journal of Orthopedic Trauma (TLS = 161,653), Injury-international Journal of The Care of The Injured (TLS = 109,816), Clinical Orthopedics And Related Research (TLS = 112,802) (Figure 7).

#### Publications

**Figure 8:**
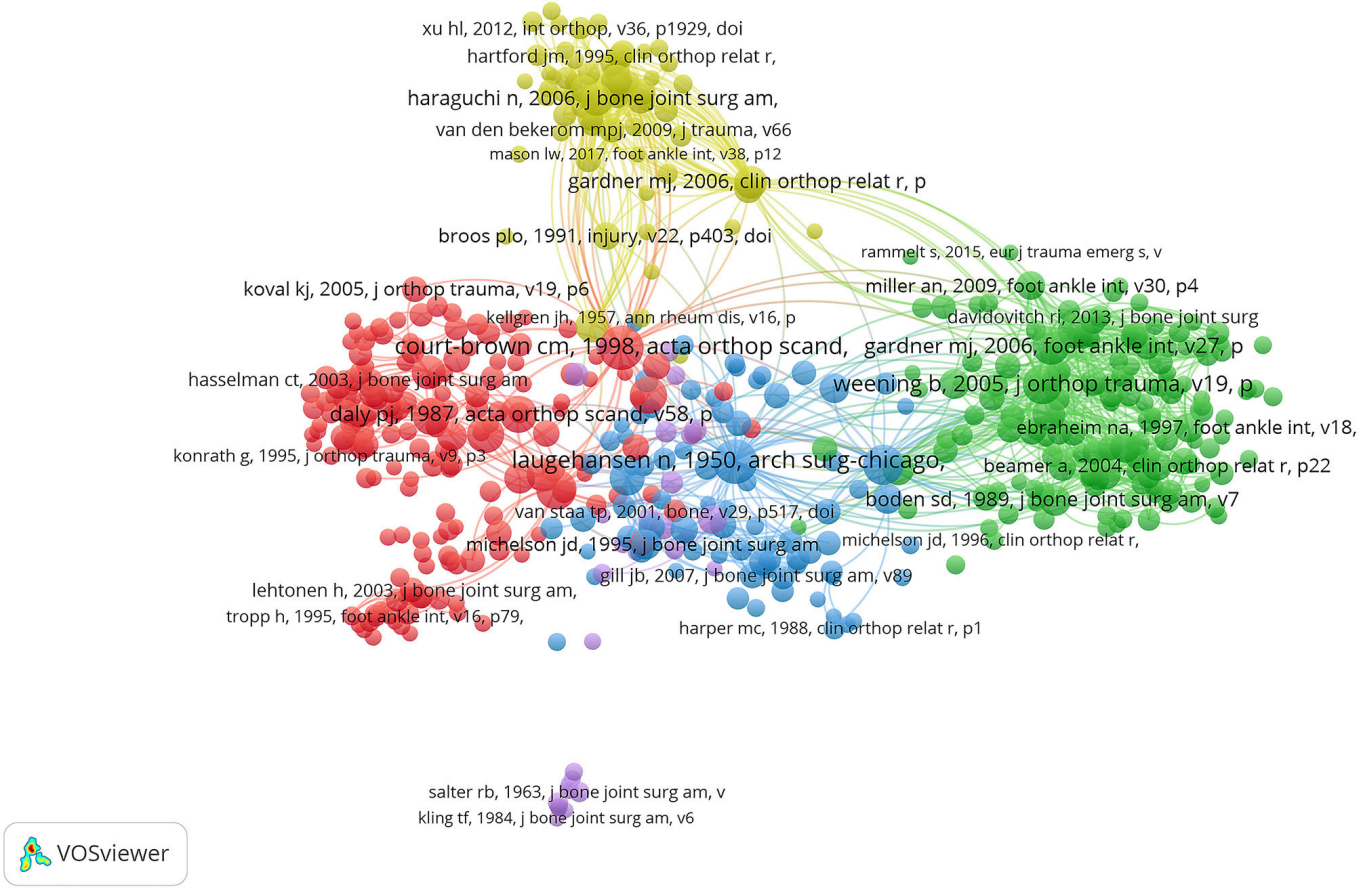
Co-citation analysis map of publications on ankle fractures.

A total of 490 articles were cited at least 20 times. The five articles with the highest number of TLS were, in order, Lauge-Hansen N (15) (TLS = 4,787), Weening B (16) (TLS = 4,410), Gardner MJ (17) (TLS = 4,023), Court-Brown CM (18) (TLS = 4,002), Ramsey PL (19) (TLS = 3,869) (Figure 8).

### Co-occurrence Analysis

A total of 332 keywords were used at least 10 times. Based on this, the keywords were classified into 7 clusters by cluster analysis.

**Figure 9:**
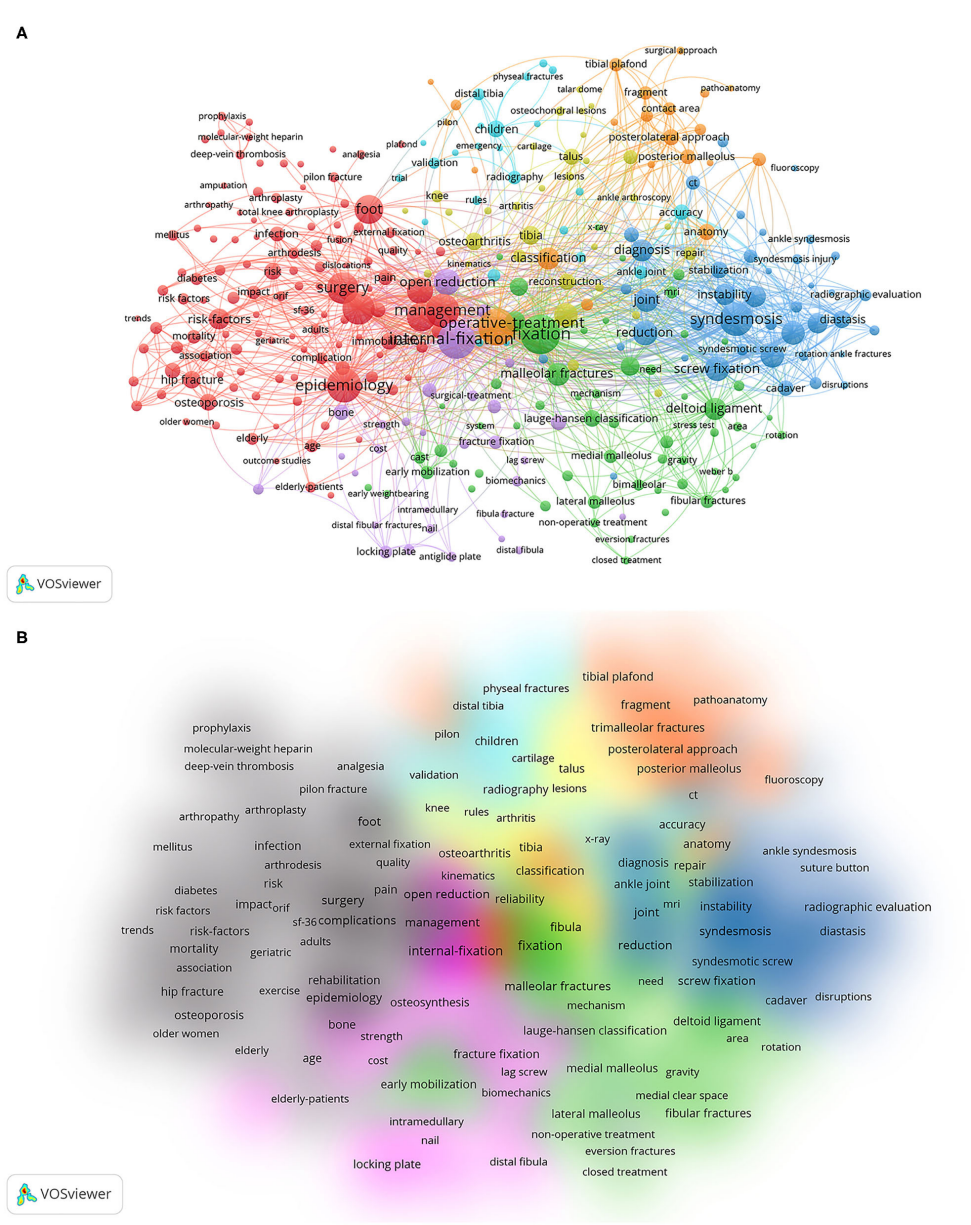
Co-occurrence analysis of global research on ankle fractures. **(A)** Keywords-mapping of co-occurrence analysis of global research on ankle fractures. **(B)** Density distribution of co-occurrence analysis of global research on ankle fractures.

1. Survival analysis and prognostic studies: epidemiology, risk-factors, complication, outcome, risk, obesity, Osteoporosis, woman, diabetes, deep-vein thrombosis, infection, pain, mortality, body-mass index, bone-mineral density, postmenopausal women.
2. Internal fixation treatment studies: internal-fixation, open reduction, osteosynthesis, plate, locking plate, antiglide plate, fibular nail, lag screw, implants, strength, functional outcomes.
3. Treatment study of combined deltoid ligament rupture: Malleolar fracture, stability, reliability, MRI, ultrasonography, stress radiography, complex, deltoid ligament, bimalleolar fracture, medial fracture, supination-eversion fracture, fibular fractures, weber b, lauge-hanse, lateral mallelus, non-operative treatment,
4. Treatment study of combined inferior tibiofibular ligament joint injury: syndesmosis, tibiofibular syndesmosis, screw fixation, diagnosis, stabilization, stabilization, joint, instability, radiographic measurements, rotational malreduction, radiographic evaluation, suture button Operative treatment, classification,
5. Treatment study of posterior ankle fractures: internal-fixtion, classification, posterior malleolar fracture, pilon, trimalleolar fracture, tibial margin, anatomy, classification
6. Treatment study of postoperative traumatic arthritis after ankle fracture: osteoarthritis, arthroscopy, reconstruction, tibia, fibula, repir, talus, inflammation, gait analysis.
7. Treatment study of ankle injuries in children: children, radiography, distal tibial epiphysis, emergency, validation, distal tibia, rules, ankle injury, accuracy (Figure 9).

**Figure 10:**
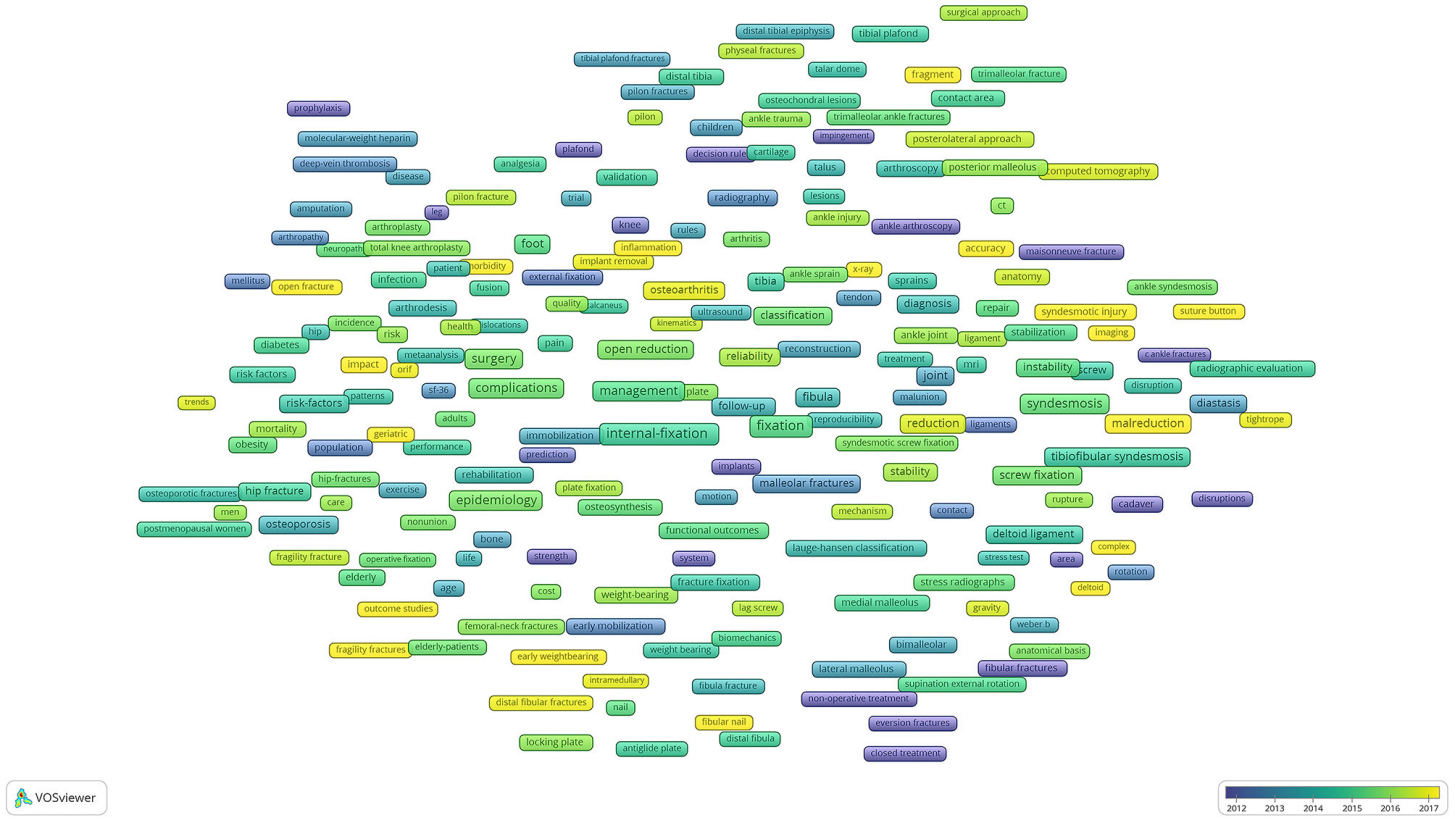
Average year map of keywords on ankle fractures.

New emerging keywords from 2013 to 2018: inflammation, fibular nail, fragility fracture, geriatric, imaging, accuracy, x-ray, suture-button, deltoid, outcome, studies, syndesmotic injury, open fracture, osteoarthritis, intromedullary, complex, weight-bearing radiography, impact, surgical site infection, fragment, pathoanatomy, tightrope fixation, suture-button, early weight-bearing, fibular, geriatric, fragility fracture, ORIF, osteoarthritis, quality of life, deltoid, MCS, distal fibular fractures, ankle instability, malreduction, posttraumatic osteoarthritis, pathoanatomy (Figure 10). After artificially judging the keyword relevance, we can classify the above terms into 4 categories as follows: (1) survival analysis and prognosis studies; (2) internal fixation treatment studies; (3) treatment study of combined deltoid ligament rupture; (4) combined inferior tibiofibular joint ligament injury.

## Discussion

### Global Trends in Ankle Fracture Research

This study provides a comprehensive overview of trends and developments in ankle fractures from 2000 to 2021. The number of articles related to ankle fractures has increased 6.7-fold since 2000, reflecting the importance that the medical community places on the economic and social burden of ankle fractures. The number of relevant publications is also projected to exceed 600 in 10 years by 2031 based on predictive models, suggesting that the research fervor related to ankle fractures will continue to be maintained and increase with the aging process.

### Status and Quality of Global Publications

H-index and total citations are important indicators of the scholarly impact and quality of publications (12). The United States has the highest number of publications and citations and the highest average citation frequency, indicating that it contributes greatly to the field. Some European countries such as the United Kingdom, Germany, the Netherlands, and Switzerland also play an important role in the field of ankle fracture research due to their high H-index and average citation frequency. In Asia, only China and South Korea are in the top 10. In contrast, most of the countries in Southeast Asia, Central Asia, Eastern Europe, and Africa have hardly contributed to this field of research, probably due to the differences in the socio-economic development of each region. The top 10 affiliations in terms of the number of publications all belong to Western countries (7 in the US and 3 in Western Europe), and also the top 10 organizations funded the most number of studies except NSFC from China and the rest from Western countries (US 6, EU 1, UK 1, Australia 1). The above results suggest that Western countries, especially the United States, still dominate the current direction of ankle fracture research, which also indicates that top-notch research institutions and adequate financial support are essential to improve the overall academic contribution of a country.

Similarly, there was a clear concentration in the statistics of published journals and published authors. The top 10 journals in terms of total published literature on ankle fractures together account for approximately one-third of all literature in the field, especially Foot & Ankle International, which accounts for more than 10%. Lorich DG, Rammelt S, Egol KA, Schepers T, Kwon JY, Helfet DL, Tornetta P, Hoogendoorn JM, Van Den Bekerom MPJ, Van Dijk CN, Van Dijk CN, and ten other authors published 10% of the total number of articles in the literature, which indicates that the above-mentioned journals and authors deserve our attention. Meaningful and groundbreaking results in the field of ankle fracture research are more likely to be published in these journals, and the studies of the above authors are more likely to reflect recent advances in the field of ankle fracture research.

However, our analysis also revealed that although the literature related to ankle fractures is increasing year by year, the absolute and relative number of high-quality RCT studies among them is still quite low (140/2923, 4.89%), and the same was true in 2020 (22/321; 7.85%). Possible reasons for this are as follows: (1) Due to different levels of economic development, registration systems and follow-up of fracture patients are incomplete in many less developed countries. (2) Although the importance of ankle fractures is increasing with the aging process, it is still not receiving enough attention.

### Research Focus on Ankle Fractures

In the first cluster, the article with the highest total link strength is *Adult Ankle Fractures-An Increasing Problem?* (TLS = 4,002), an epidemiological survey of ankle fractures published in 1998 by Court-Brown (18) in *Acta Orthop Scand*, which included outpatients in addition to inpatients as well as more realistic results than other similar epidemiological studies of ankle fractures of the same period. Simultaneously, it is also the first article to classify a large series of ankle fractures into its constituent A0 subgroups. It is concluded by the study that the population with the highest incidence of ankle fractures with increasing longevity was women between the ages of 75 and 84 years, and the typing results showed that type 44-a accounted for 38% of the total amount, type 44-b for 52%, and type 44-c for 10%.

In the second cluster, the article with the highest total link strength is *Predictors Of Functional Outcome Following Transsyndesmotic Screw Fixation Of Ankle Fractures* (TLS = 4,410), a retrospective observational study published in 2005 by Brad Weening and Mohit Bhandari (16) in *J Orthop Trauma* (Level IV). The prognosis of a total of 51 patients with ankle injuries who received syndesmotic screw fixation at 3 university-affiliated hospitals from 1998 to 2001 was reviewed in the study and the following results were achieved: (1) 16% of syndesmotic screws may have been unnecessary; (2) The anatomic reduction of syndesmosis was remarkably associated with enhanced Short Musculoskeletal Functional Assessment Index functional outcome.

In the third cluster, the most cited article with the highest total link strength is *Changes In Tibiotalar Area Of Contact Caused By Lateral Talar Shift* (TLS = 3,869), a biomechanical study published in 1976 by Ramsey and Hamiliton (19) in *The Journal of Bone* & *Joint Surgery*. It is suggested in this article that a lateral displacement of the talus by 1 mm reduces the contact area of the ankle joint by 42%, which in turn leads to uneven pressure distribution in the ankle joint and thereby causes traumatic ankle arthritis. It has been shown by the existing study that the deltoid ligament prevents external rotation of the talus and thus plays a vital role in maintaining the stability of the ankle joint (20, 21). Combined with the study of Ramsey and Hamiliton, it is concluded that ankle fractures with deltoid ligament injury should be treated with aggressive ligament repair therapy.

In the fourth cluster, the article with the highest total link strength is A*natomy of the Distal Tibiofibular Syndesmosis in Adults: a pictorial essay with a multimodality approach* (TLS = 990), published in 2010 by Hermans (22) in *J Anat*. The description of the anatomy of the separate osseoligamentous structures of the distal tibiofibular joint and discussion of the clinical relevance of these structures were carried out in the above-mentioned anatomical overview, thus it can improve the orthopedic surgeon's understanding and knowledge of anatomy, imaging as well as surgical treatment of distal tibiofibular syndesmosis in adults.

In the fifth cluster, the article with the highest total link strength is *Effect of Posterior Malleolus Fracture on Outcome after Unstable Ankle Fracture* (TLS = 1,634), a prospective study published in 2010 by Tejwani (23) in *J Trauma*, and the prognosis of 309 patients with unstable ankle fractures treated operatively (255 patients without posterior ankle fracture vs. 54 patients with posterior ankle fracture) was followed up. The results showed improvements in function and pain status in all patients at 12 months follow-up, but patients with posterior malleolus fractures had significantly worse total scores and pain function according to the American Orthopedic Foot and Ankle Society score. Similar conclusions were reached on the Short Form-36 questionnaire and the Short Musculoskeletal Function Assessment questionnaire, which were statistically different when the posterior malleolar fracture group scored lower than the unaccompanied posterior malleolar fracture group. It is hereby concluded that patients with posterior malleolus fractures have a worse prognosis.

In the sixth cluster, the article with the highest total link strength is *Posttraumatic Ankle Osteoarthritis after Ankle-Related Fractures* (TLS = 945), published in 2009 by Monika Horisberger (24) in *J Orthop Trauma*, a retrospective cohort study on 141 patients with a history of ankle-related fractures. This is the first study to analyze the etiology, mechanisms, and risk factors of posttraumatic ankle osteoarthritis (OA). The results showed that ankle fractures were the most common OA-related fractures, accounting for 53.2%. The mean time from OA-related injury to end-stage ankle OA was 20.9 years (1–52 years). The mean time between injury onset and progression to end-stage ankle OA is 20.9 years (1–52 years). OA latency is remarkably shorter in patients with complications during fracture healing and there is a negative correlation between age (at the time of ankle injury) and OA latency.

In the seventh cluster, the article with the highest total link strength is *A Randomized, Controlled Trial of a Removable Brace vs. Casting in Children with Low-Risk Ankle Fractures* (TLS = 288), a randomized, single-blind trial published by Kathy Boutis (25) in *Pediatrics*. The above-mentioned study compared the outcomes of 104 children aged 5–18 years with isolated distal fibular ankle fractures treated with a removable ankle brace (*n* = 54) or casting (*n* = 50) and it was shown by the results that plaster fixation remains the standard treatment, but the mean activity score at 4 weeks in the brace group was significantly higher than the score in the cast group (91.3 vs. 85.3%). And the cost-effectiveness acceptability curve was invariably shown to be >80%. Concerning the recovery of physical function, the removable ankle brace is excel in its effectiveness when compared with the cast and is cost-effective as well.

The overlay visualization map showed that the hot spots in ankle fracture research in recent years were survival and prognosis studies, treatment of combined tibiofibular joint ligament injury, internal fixation treatment, and treatment of combined deltoid ligament rupture, which emerged later than the research directions, indicating that there is great potential for follow-up research and researchers can continue to explore these directions in the future.

## Limitations

The limitations of this study are: (1) The literature included is from one database, which may lead to the inclusion of a small number of articles for analysis compared to the number of real relevant articles; (2) Only English published literature is included, which may lead to language bias and ignore the contribution of non-English literature to the development of the discipline as a whole; (3) Some of the best articles published in recent years, due to their low citation frequency, may also lead to errors in the results.

## Conclusions

This study illustrates the current state of global research on ankle fractures between 2000 and 2021 and predicts future research trends in this field. Foot & Ankle International can be considered a landmark journal since it has published the highest number of citations on ankle fractures. The survival and prognosis analysis, the treatment of combined tibiofibular ligament injuries, the treatment of internal fixation, and the treatment of combined deltoid ligament ruptures will be a hot topic of research in the future. We also need to collaborate to conduct more high-quality RCT studies. In addition, we call for the establishment of standardized ankle fracture registry follow-up in Southeast Asian, Eastern European, and African countries as soon as possible, which will help to further improve the global system of ankle fracture management.

## Data Availability Statement

The raw data supporting the conclusions of this article will be made available by the authors, without undue reservation.

## Author Contributions

JZ: conceptualization, methodology, software, validation, formal analysis, data curation, writing–original draft, and visualization. JL: conceptualization, methodology, software, validation, and writing–review and editing. GX, WZ, DW, HL, XG, and YX: data curation. CX: visualization and supervision. LZ: project administration and writing–review and editing. PT: project administration, writing–review and editing, and funding acquisition. All authors contributed to the article and approved the submitted version.

## Funding

This work was supported by The 13th Five-year Plan for Key Discipline Construction Project of PLA (A350109).

## Conflict of Interest

The authors declare that the research was conducted in the absence of any commercial or financial relationships that could be construed as a potential conflict of interest.

## Publisher's Note

All claims expressed in this article are solely those of the authors and do not necessarily represent those of their affiliated organizations, or those of the publisher, the editors and the reviewers. Any product that may be evaluated in this article, or claim that may be made by its manufacturer, is not guaranteed or endorsed by the publisher.
